# Identification of a Novel Ceftazidime-Avibactam-Resistant KPC-2 Variant, KPC-123, in *Citrobacter koseri* Following Ceftazidime-Avibactam Treatment

**DOI:** 10.3389/fmicb.2022.930777

**Published:** 2022-06-20

**Authors:** Lin Wang, Weiyi Shen, Rong Zhang, Jiachang Cai

**Affiliations:** Clinical Microbiology Laboratory, The Second Affiliated Hospital of Zhejiang University School of Medicine, Zhejiang University, Hangzhou, China

**Keywords:** inhibitor resistance, KPC variant, OXA-232, enterobacterales, antibiotic treatment

## Abstract

This study reported the identification of a novel ceftazidime-avibactam-resistant KPC-2 variant, KPC-123, in a *Citrobacter koseri* isolated from a patient in a Chinese hospital following ceftazidime-avibactam treatment of infection caused by OXA-232-producing *Klebsiella pneumoniae*. This novel KPC-123 consisting of 302 amino acids differs from KPC-2 by two insertions after positions 179 (ins179_TY) and 270 (ins270_DDKHSEA), respectively. Conjugation and cloning experiments confirmed that KPC-123 was able to confer high-level resistance to ceftazidime and ceftazidime/avibactam (MICs of 128 mg/L and 64/4 mg/L, respectively) and elevated MIC values of cefotaxime, cefepime, and aztreonam (4 mg/L, 2 mg/L, and 4 mg/L, respectively) but retained susceptibility to carbapenems. Whole-genome sequencing and genomic analysis revealed that *bla*_KPC−123_ within the “IS*Kpn27*-*bla*_KPC_-IS*Kpn6*” structure was located on a 93,814-bp conjugative plasmid that was almost identical to a *bla*_KPC−2_-carrying plasmid harbored in a *K. pneumoniae* isolate from the same sampling site of the patient, suggesting the transfer and *in vivo* evolution of this *bla*_KPC_-carrying plasmid. Hence, active surveillance of ceftazidime/avibactam resistance and the underlying mechanisms, which may facilitate the prevention and control of the dissemination of resistance, is needed.

## Introduction

Carbapenem-resistant enterobacterales (CRE), especially for *Klebsiella pneumoniae* (CRKP), are spreading worldwide and pose a serious threat to the public health (Wang et al., 2022). According to the data from the China Antimicrobial Surveillance Network (CHINET), the rate of CRKP in the Chinese tertiary hospitals had dramatically increased from 3.0% in 2005 to 26.3% in 2018 (Hu et al., 2019). Another nationwide survey in China showed that the production of KPC-2 carbapenemase (accounting for 74%) was the predominant mechanism of carbapenem resistance in CRKP (Zhang et al., 2017). Ceftazidime-avibactam, a novel β-lactam/β-lactamase inhibitor combination, is active against enterobacterales-producing KPC or OXA-48-like carbapenemase (van Duin and Bonomo, 2016) and is currently considered as one of the last-line antimicrobial agents for the treatment of infections involving these strains (Sheu et al., 2019). However, resistance to ceftazidime-avibactam in *K. pneumoniae* has begun to emerge shortly after its approval in 2015 (Shields et al., 2017). Amino acid substitutions in the KPC enzyme were the main factor responsible for ceftazidime-avibactam resistance, though other mechanisms such as increased *bla*_KPC_ gene expression and/or decreased membrane permeability were involved (Wang et al., 2020).

To date, more than 100 KPC variants have been identified, about one-third of which (38/113) were resistant to ceftazidime-avibactam (https://www.ncbi.nlm.nih.gov/pathogens/refgene/#KPC). Mutations in the Ω-loop (at Arg_164_ to Asp_179_ in Ambler numbering) which is proximal to the KPC active site, especially D179Y substitution in KPC-2 (KPC-33) and KPC-3 (KPC-31), were initially described and seemed to be the most common type conferring resistance to ceftazidime-avibactam (Compain and Arthur, 2017; Shields et al., 2017; Wang et al., 2020). Other point mutations, insertions, and deletions can also be observed in the Ω-loop and two additional regions (at Cys_238_ to Thr_243_ and Ala_267_ to Ser_275_, respectively) of KPC from both clinical isolates and ceftazidime-avibactam-resistant mutants selected *in vitro* (Hobson et al., 2020; Wang et al., 2020; Venditti et al., 2021). In total, three inhibitor-resistant KPC variants derived from KPC-2, KPC-33, KPC-71, and KPC-74, were recently identified in *K. pneumoniae* isolates from China (Shi et al., 2020; Li et al., 2021b; Shen et al., 2022). Here, we described the *in vivo* emergence and evolution of a novel ceftazidime-avibactam-resistant KPC-2 variant, KPC-123, in a clinical isolate of *Citrobacter koseri* following ceftazidime-avibactam treatment of intracranial infection caused by OXA-232-producing CRKP.

## Materials and Methods

### The Patient and Bacterial Strains

A 67-year-old male with cerebellar hemorrhage underwent emergency surgery and was admitted to the neurosurgical intensive care unit (NICU) of a tertiary hospital in Hangzhou, China, in 2021. Empirical combination therapy with imipenem (0.5 g IV every 6 h) plus linezolid (0.6 g IV every 12 h) was started (from day 2 to day 13) due to the persistent fever after the operation. In total, 3 days after admission to the hospital, a carbapenem-resistant *Acinetobacter baumannii* (CRAB) emerged in the patient's sputum sample and had not been cleared until he was discharged. Two CRKP (strains WS420 and SP422) were isolated from wound secretion and sputum cultures on the 13th and 15th days, respectively. Thus, tigecycline was used (0.1 g IV every 12 h) instead of the previous antimicrobials for 8 days (from day 14 to day 21) and the patient's temperature became normal. In total, 4 days after treatment ended, the patient developed fever again. Both the blood culture and cerebrospinal fluid culture were performed and the latter grew a CRKP (strain CF503) on day 26. The culture of the rectal swab showed colonization by a CRKP (strain RS503). Polymyxin B (50,000 IU IT once daily) combined with ceftazidime-avibactam (2.5 g IV every 8 h) were administrated according to the antimicrobial susceptibility result of *K. pneumoniae* CF503. Polymyxin B was withdrawn after 1 week of treatment (day 27 to day 33) when the patient's intracranial infection was controlled. The subsequent cultures of cerebrospinal fluid and sputum showed a negative result for CRKP but reported the new growth of *Burkholderia cepacia* in the sputum sample. In the following hospitalization, the patient accepted multiple operations because of obstructive hydrocephalus, including lumbar cistern drainage, external ventricular drainage, Ommaya reservoir implantation, and ventriculoperitoneal shunt. To prevent the potential nosocomial infection, ceftazidime-avibactam was maintained. After 30 days of ceftazidime-avibactam administration (day 56), a *C. koseri* (strain CK1008) exhibited ceftazidime-avibactam resistance but retained susceptibility to carbapenems was isolated from the sputum sample. Considering that the patient was stable and afebrile, no additional antimicrobial was added and the patient was discharged from the hospital on day 72.

To understand the mechanisms and evolutionary route of ceftazidime-avibactam resistance, *Citrobacter koseri* CK1008 and four CRKP (strains WS420, SP422, CF503, and RS503) isolated from different samples of the same patient (Table 1) were subjected to whole-genome sequencing (WGS) and further analysis. This study was approved by the Ethics Committee of The Second Affiliated Hospital of Zhejiang University School of Medicine and consent was given by the patient.

**Table 1:** Characteristics and carriage of antibiotic resistance genes of *C. koseri* CK1008 and four *K. pneumoniae* isolates.

**Strain**	**Specimen**	**Sequence type**	**Antibiotic resistance genes**	**Conjugation efficiency of carbapenem gene**	**Mutation in *ompK* genes**	**Virulence factors**
*K. pneumoniae* WS420	Wound secretion	ST15	*bla*_OXA−232_, *bla*_SHV−28_, *bla*_CTX−M−15_, *fosA, qnrB1, oqxAB, rmtF, aac(6')-Ib, dfrA14, ARR-2*	Non-conjugative	Truncated *ompK35*	*rmpA2*, Aerobactin, Yersiniabactin
*K. pneumoniae* SP422	Sputum	ST11	*bla*_KPC−2_, *bla*_CTX−M−65_, *bla*_SHV−12_, *bla*_TEM−1_, *fosA, aadA2b, rmtB*	4.0 ×10^−2^	Truncated *ompK35* and *ompK37*; absence of *ompK36*	*ramA, rmpA, rmpA2*, Aerobactin, Yersiniabactin
*K. pneumoniae* CF503	Cerebrospinal fluid	ST15	*bla*_OXA−232_, *bla*_SHV−28_, *bla*_CTX−M−15_, *fosA, qnrB1, oqxAB, rmtF, aac(6')-Ib, dfrA14, ARR-2*	Non-conjugative	truncated *ompK35*	*rmpA2*, Aerobactin, Yersiniabactin
*K. pneumoniae* RS503	Rectal swab	ST15	*bla*_OXA−232_, *bla*_SHV−28_, *bla*_CTX−M−15_, *fosA, qnrB1, oqxAB, rmtF, aac(6')-Ib, dfrA14, ARR-2*	Non-conjugative	Truncated *ompK35*	*rmpA2*, Aerobactin, Yersiniabactin
*C. koseri* CK1008	Sputum	/	*bla*_KPC−123_, *bla*_CKO_	1.5 ×10^−2^	/	/

### Antimicrobial Susceptibility Testing

The minimal inhibitory concentrations (MICs) of 15 antimicrobial agents, including imipenem, meropenem, ertapenem, ceftazidime, cefotaxime, cefepime, piperacillin/tazobactam, cefoperazone/sulbactam, aztreonam, cefmetazole, ciprofloxacin, amikacin, tigecycline, colistin, and ceftazidime/avibactam, were determined using the broth microdilution method (Clinical Laboratory Standards Institute, 2018). The results were interpreted according to the CLSI recommendations (Clinical and Laboratory Standards Institute, 2021). *Escherichia coli* ATCC 25922, *K. pneumoniae* 700603, and *Pseudomonas aeruginosa* ATCC 27853 were used as the quality control strains in parallel. The susceptibility breakpoint for cefoperazone was applied for cefoperazone/sulbactam. Tigecycline susceptibility was interpreted using breakpoints recommended by the US Food and Drug Administration (https://www.fda.gov/drugs/development-resources/tigecycline-injection-products).

### Whole-Genome Sequencing and Genome Analysis

Genomic DNA extracted from *C. koseri* CK1008 and four *K. pneumoniae* (strains WS420, SP422, CF503, and RS503) were subjected to WGS using the Illumina NovaSeq 6000 platform. The reads were *de novo* assembled with SPAdes v.3.13.1 (Bankevich et al., 2012). The sequence types and carriage of antimicrobial resistance genes were identified at the Center for Genomic Epidemiology (CGE) (http://www.genomicepidemiology.org/services/) using MLST 2.0 and ResFinder 4.1, respectively (Larsen et al., 2012; Bortolaia et al., 2020). The plasmid types were identified by using PlasmidFinder 2.1 available at CGE (Carattoli et al., 2014). A comparison of sequences of *bla*_KPC_-carrying plasmids was conducted using BRIG (v0.95) (Alikhan et al., 2011). Virulence genes were identified using Kleborate (v0.3.0). The genetic relatedness among CRKP isolates producing KPC or OXA-232 was investigated by single-nucleotide polymorphism (SNP) typing. Core-genome alignment, SNP calling, and the maximum likelihood phylogeny were constructed using the harvest suite including Parsnp (Treangen et al., 2014). The generated phylogenetic tree was edited and visualized by iTOL (v3) (Letunic and Bork, 2016). The contigs containing the *bla*_KPC−123_ gene were aligned with the database of GenBank using the BLASTN program and the putative gaps were filled by PCRs and Sanger sequencing according to the sequence of the reference plasmid.

### Conjugation and Molecular Cloning Experiments

To evaluate the transferability of *bla*_KPC_-carrying plasmids, conjugation experiments were performed with filter mating methods. Rifampin-resistant *E. coli* EC600 was used as the recipient strain and the known *bla*_KPC−2_-positive *K. pneumoniae* K1 was used as the positive control strain (Cai et al., 2008). The putative transconjugants that grew on agar plates containing 500 mg/L rifampin and 0.3 mg/L meropenem (for KPC-2-producers) or 4 mg/L ceftazidime-avibactam (for KPC-123 producers) were identified by MALDI-TOF MS and screened for the *bla*_KPC_ gene by PCR. The conjugation frequency was calculated as the ratio of the number of transconjugants to the number of donors. To check whether the KPC-123 β-lactamase contributes to the ceftazidime-avibactam resistance phenotype, the DNA fragment containing the *bla*_KPC−123_ gene, and its putative promoter was amplified by PCR using primers (5′-CGCGGATCCCTCCAACACAAAACACCCGT-3′) and (5′-CCCAAGCTTGCG CAGACTCCTAGCCTAAA-3′) that contained introduced BamHI and HindIII restriction sites (underlined), respectively. Amplicons were digested with BamHI and HindIII (ThermoFisher scientific, Lithuania) and ligated to cloning vector pHSG396 (TaKaRa, Dalian, China) digested with the same restriction enzymes. The recombinant plasmid was transformed to *E. coli* DH5α. The transformants were selected on the plate containing rifampin and ceftazidime-avibactam. The inserted fragment was amplified by PCR and double-stranded sequencing was performed to ensure that no mutation was introduced.

### Fitness of the *E. coli* Recipient Carrying the *Bla*_KPC-123_ Gene

The fitness of *E. coli* EC600 was assessed by plotting the growth curves for both the transconjugants and the recipient in triplicate. Individual strains were grown exponentially in Luria–Bertani (LB) broth with shaking (200 rpm) at 37°C to an optical density at 600 nm (OD600) of 1. In total 100 μl of the culture were inoculated to the subculture with 10 ml of fresh broth and then incubated with shaking at 37°C. OD600 measurements were recorded at intervals of 1 h, and finally plotted as a growth curve using the GraphPad Prism 7.0 software.

### Nucleotide Sequence Accession Numbers

The genomes of *C. koseri* CK1008 and four *K. pneumoniae* (WS420, SP422, CF503, and RS503) have been deposited in the NCBI database under BioProject accession number PRJNA823947 and GenBank accession numbers JALNMB000000000, JALNMF000000000, JALNME000000000, JALNMD000000000, and JALNMC000000000. The complete sequence of the plasmid pCK1008-KPC-123 has been deposited in the NCBI database under GenBank accession number ON209376.

## Results

### Antimicrobial Susceptibility Results

As shown in Table 2, *C. koseri* CK1008 was high-level resistant to ceftazidime and ceftazidime/avibactam (MICs of >128 and 64/4 mg/L, respectively) and was low-level resistant to cefotaxime and cefepime (MICs of 8 and 16 mg/L, respectively), but was susceptible to carbapenems and other tested antibiotics. In total, four *K. pneumoniae* isolate exhibited a similar susceptibility profile except for the much higher MICs of carbapenems, aztreonam, and cefmetazole in strain SP422. All *K. pneumoniae* isolates were susceptible to ceftazidime/avibactam, tigecycline, and colistin.

**Table 2:** Antimicrobial susceptibility results of *C. koseri* CK1008 and four *K. pneumoniae* isolates and their *E. coli* transconjugants and transformants.

**Strain**	**MICs (mg/L)**														
	**IPM^**a**^**	**MEM**	**ETP**	**CAZ**	**CTX**	**FEP**	**TZP**	**SCF**	**ATM**	**CMZ**	**CIP**	**AK**	**TGC**	**COL**	**CZA**
*K. pneumoniae* WS420	4	8	64	64	>128	>64	>256/4	>256/128	64	32	>32	>128	0.5	≤ 0.5	≤ 0.5/4
*K. pneumoniae* SP422	64	128	>128	>128	>128	>64	>256/4	>256/128	>128	>128	>32	>128	0.5	≤ 0.5	1/4
*K. pneumoniae* CF503	4	8	64	64	>128	>64	>256/4	>256/128	64	32	>32	>128	0.5	≤ 0.5	≤ 0.5/4
*K. pneumoniae* RS503	4	8	64	64	>128	>64	>256/4	>256/128	128	32	>32	>128	0.5	≤ 0.5	≤ 0.5/4
*C. koseri* CK1008	≤ 0.5	≤ 0.5	≤ 0.5	>128	8	16	≤ 8/4	≤ 8/4	4	4	≤ 0.25	≤ 4	≤ 0.25	≤ 0.5	64/4
*E. coli* EC600	≤ 0.5	≤ 0.5	≤ 0.5	≤ 1	≤ 1	≤ 1	≤ 8/4	≤ 8/4	≤ 1	≤ 2	≤ 0.25	≤ 4	≤ 0.25	1	≤ 0.5/4
*E. coli* EC600 transconjugant of *K. pneumoniae* SP422	2	4	8	16	16	8	256/4	64/32	128	8	≤ 0.25	≤ 4	≤ 0.25	≤ 0.5	≤ 0.5/4
*E. coli* EC600 transconjugant of *C. koseri* CK1008	≤ 0.5	≤ 0.5	≤ 0.5	128	4	2	≤ 8/4	≤ 8/4	4	≤ 2	≤ 0.25	≤ 4	≤ 0.25	≤ 0.5	64/4
*E. coli* DH5α	≤ 0.5	≤ 0.5	≤ 0.5	≤ 1	≤ 1	≤ 1	≤ 8/4	≤ 8/4	≤ 1	≤ 2	≤ 0.25	≤ 4	≤ 0.25	≤ 0.5	≤ 0.5/4
*E. coli* DH5α/pHSG396	≤ 0.5	≤ 0.5	≤ 0.5	≤ 1	≤ 1	≤ 1	≤ 8/4	≤ 8/4	≤ 1	≤ 2	≤ 0.25	≤ 4	≤ 0.25	≤ 0.5	≤ 0.5/4
*E. coli* DH5α/pHSG396-KPC-123	≤ 0.5	≤ 0.5	≤ 0.5	64	4	2	≤ 8/4	≤ 8/4	4	≤ 2	≤ 0.25	≤ 4	≤ 0.25	≤ 0.5	32/4

*^a^IPM, imipenem; MEM, meropenem; ETP, ertapenem; CAZ, ceftazidime; CTX, cefotaxime; FEP, cefepime; TZP, piperacillin/tazobactam; SCF, cefoperazone/sulbactam; ATM, aztreonam; CMZ, cefmetazole; CIP, ciprofloxacin; AK, amikacin; TGC, tigecycline; COL, colistin; CZA, ceftazidime/avibactam. For piperacillin/tazobactam and ceftazidime/avibactam, the tazobactam and avibactam were tested at a fixed concentration of 4 mg/L. For cefoperazone/sulbactam, the combination was tested with concentrations of 2:1 ratio (antibiotic: inhibitor)*.

### Genomic Analysis of KPC- and OXA-232-Producing Isolates

Screening of antimicrobial resistance determinants based on the WGS data showed that *C. koseri* CK1008 harbored a novel variant of KPC-2 and a chromosomal class A β-lactamase CKO. The novel KPC-2 variant, designated KPC-123, contains 302 amino acids which differ from KPC-2 (containing 293 amino acids) by two insertions after Ambler positions 179 (ins179_TY) and 270 (ins270_DDKHSEA), respectively. ^****^The CKO, which was identified in *C. koseri* in 2006, could mediate resistance to amoxicillin and ticarcillin (Petrella et al., 2006). Three *K. pneumoniae* (strains WS420, CF503, and RS503), which belonged to sequence type (ST) 15, possessed the same set of resistance determinants conferring resistance to β-lactams including carbapenems (*bla*_OXA−232_, *bla*_SHV−28_, and *bla*_CTX−M−15_), fosfomycin (*fosA*), quinolones (*qnrB*), aminoglycosides (*rmtF* and *aac(6')-Ib*), rifampicin (*ARR-2*), trimethoprim (*dfrA14*), and the multidrug resistance efflux pump OqxAB. The *K. pneumoniae* SP422, which was isolated from the sputum sample, belonged to ST11 and contained *bla*_KPC−2_, *bla*_CTX−M−65_, *bla*_SHV−12_, *bla*_TEM−1_, *fosA, aadA2b*, and *rmtB* genes. Sequence analysis of genes encoding outer membrane proteins revealed an internal stop codon in the *ompK35* gene (leading to termination in position 63) of four *K. pneumoniae* isolates. Premature termination of translation within the *ompK37* gene (K251^*^) and a loss of the *ompK36* gene were found in the KPC-2-producing *K. pneumoniae* SP422 which exhibited high-level resistance to carbapenems (Tables 1, 2).

**Figure 1:**
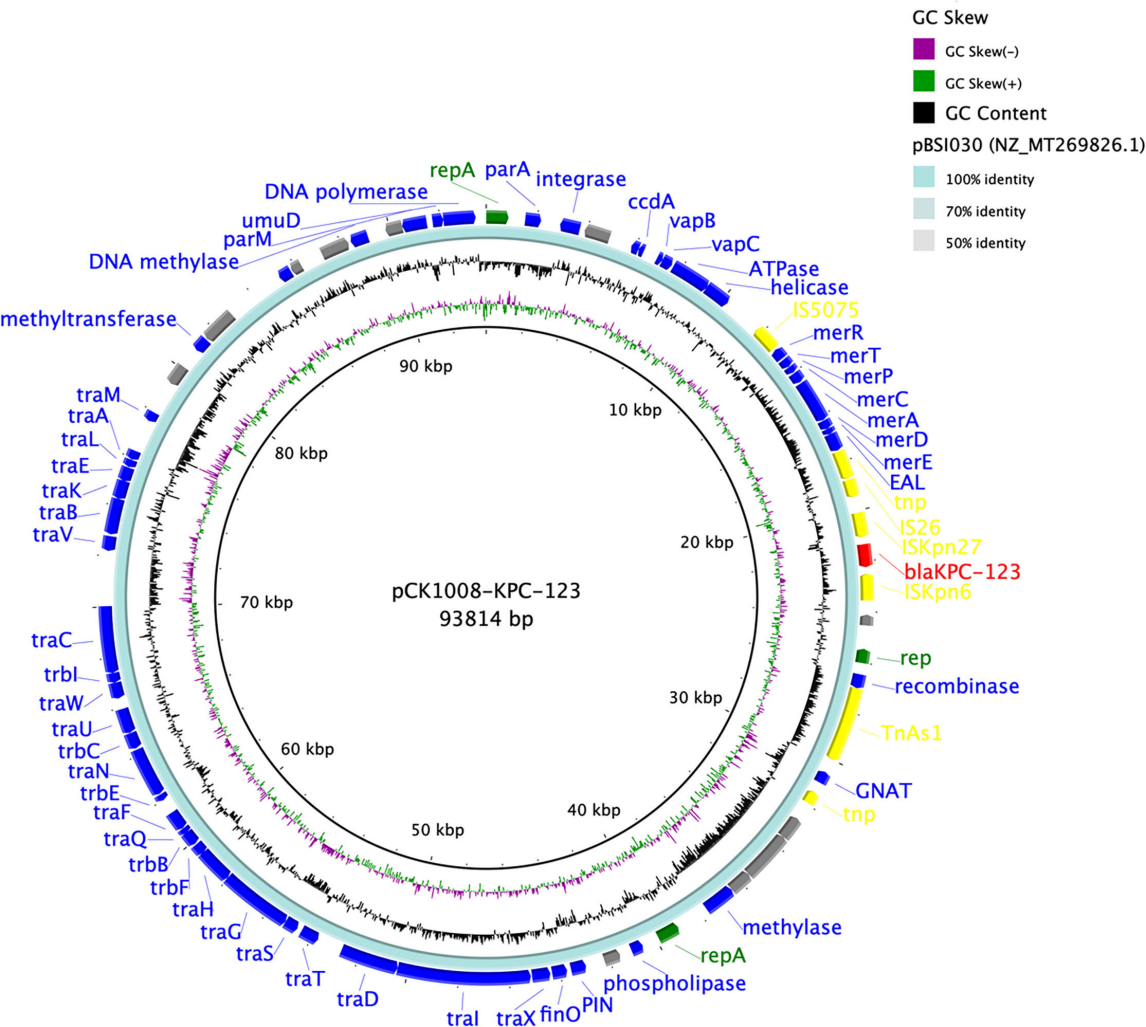
Comparison of the *bla*_KPC−123_-carrying plasmid pCK1008-KPC-123 with the reference plasmid. There were five circles from the inner to the outside that displayed the scale in kilobase pairs, the GC skew, the GC content, the similarity of the reference plasmid, and the annotation of the plasmid in our study, respectively. The antimicrobial resistance genes were labeled as red and the mobile genetic elements were labeled as yellow.

We obtained a circular 93,814 bp plasmid (pCK1008-KPC-123), which belonged to an unknown Inc-type, from *C. koseri* CK1008 by BLASTN and the gap-filling. An almost identical plasmid, which differed only by the mutations within the *bla*_KPC_ gene, was identified in *K. pneumoniae* SP422. BLASTN result showed that plasmid pCK1008-KPC-123 closely matched several *bla*_KPC−2_-carrying plasmids harbored in multiple species of Enterobacterales isolated from China, including *K. pneumoniae* (GenBank accession number MT269826.1), *Serratia marcescens* (MN823984.1), and *E. coli* (CP021195.1). The *bla*_KPC_ gene was located in a genetic structure of “IS*Kpn27*-*bla*_KPC_-IS*Kpn6*” and a large number of genes encoding proteins related to conjugative transfer were found in plasmid pCK1008-KPC-123 (Figure 1), suggesting the transferability of this plasmid and explaining its widely spread in China.

To investigate the clonal relationship among CRE isolates, Pairwise SNP analysis was performed based on the WGS data from four *K. pneumoniae* isolates in this study and 60 previously reported OXA-232-producing *K. pneumoniae* isolates from five hospitals in three cities of Zhejiang province from 2018 to 2021 (Shu et al., 2019) (Supplementary Figure 1). The core genomes of OXA-232-producers, excepting five isolates from Jiaxing city and one isolate from Hangzhou city, differed by a few SNPs with the number ranging from 1 to 89, suggesting the clonal dissemination of OXA-232-producing *K. pneumoniae* in Zhejiang province and the three isolates in this study belonged to the predominant clone. The KPC-2-producing *K. pneumoniae* SP422, however, was clonally unrelated to OXA-232-producing isolates with the SNPs number of ≥32229.

Virulence analysis showed that *K. pneumoniae* SP422 belonged to the KL64 serotype, which was the most common serotype among KPC-2-producing *K. pneumoniae* in China (Zhang et al., 2020), while the three OXA-232-producing isolates belonged to KL112. Similar to previously reported OXA-232-producers isolated from the same city (Shu et al., 2019), *K. pneumoniae* WS420, CF503, and RS503 harbored multiple genes encoding the regulators of mucoid phenotype (*rmpA2* gene) and aerobactin (*iucABCDiutA* gene cluster), which were frequently associated with hypervirulent phenotype in *K. pneumoniae* (Russo and Marr, 2019). In addition to the above virulence factors, *K. pneumoniae* SP422 produced an additional *rmpA* gene. Thus, these four isolates were considered carbapenem-resistant hypervirulent *K. pneumonia*e (CR-hvKP).

### Conjugation and Molecular Cloning Experiments

Both the *bla*_KPC_-2- and *bla*_KPC_-123-carrying plasmids could be transferred into *E. coli* EC600 from *K. pneumoniae* SP422 and *C. koseri* CK1008 with similar conjugation efficiencies of 4.0 × 10^−2^ and 1.5 × 10^−2^, respectively. However, the *bla*_OXA−232_-carrying plasmid in *K. pneumoniae* WS420, CF503, and RS503 was not transferable, which was consistent with the previous study (Shu et al., 2019). The KPC-2-producing *E. coli* transconjugant exhibited resistance to β-lactams including carbapenems but was susceptible to ceftazidime/avibactam. Conversely, the KPC-123-producing *E. coli* transconjugant developed high-level resistance to ceftazidime and ceftazidime/avibactam (MICs of 128 and 64/4 mg/L, respectively) and elevated MIC values of cefotaxime, cefepime, and aztreonam (4, 2, and 4 mg/L, respectively) but retained susceptibility to carbapenems. This antibiotic susceptibility phenotype can also be observed in *E. coli* DH5α transformant carrying the recombinant plasmid pHSG396-KPC-123 and the donor strain *C. koseri* CK1008 (Table 2). These results demonstrated that the novel KPC-123 was able to confer resistance to ceftazidime/avibactam.

### Fitness Cost of the *Bla*_KPC-2_-Carrying or *Bla*_KPC-123_-Carrying Plasmid in *E. coli*

**Figure 2:**
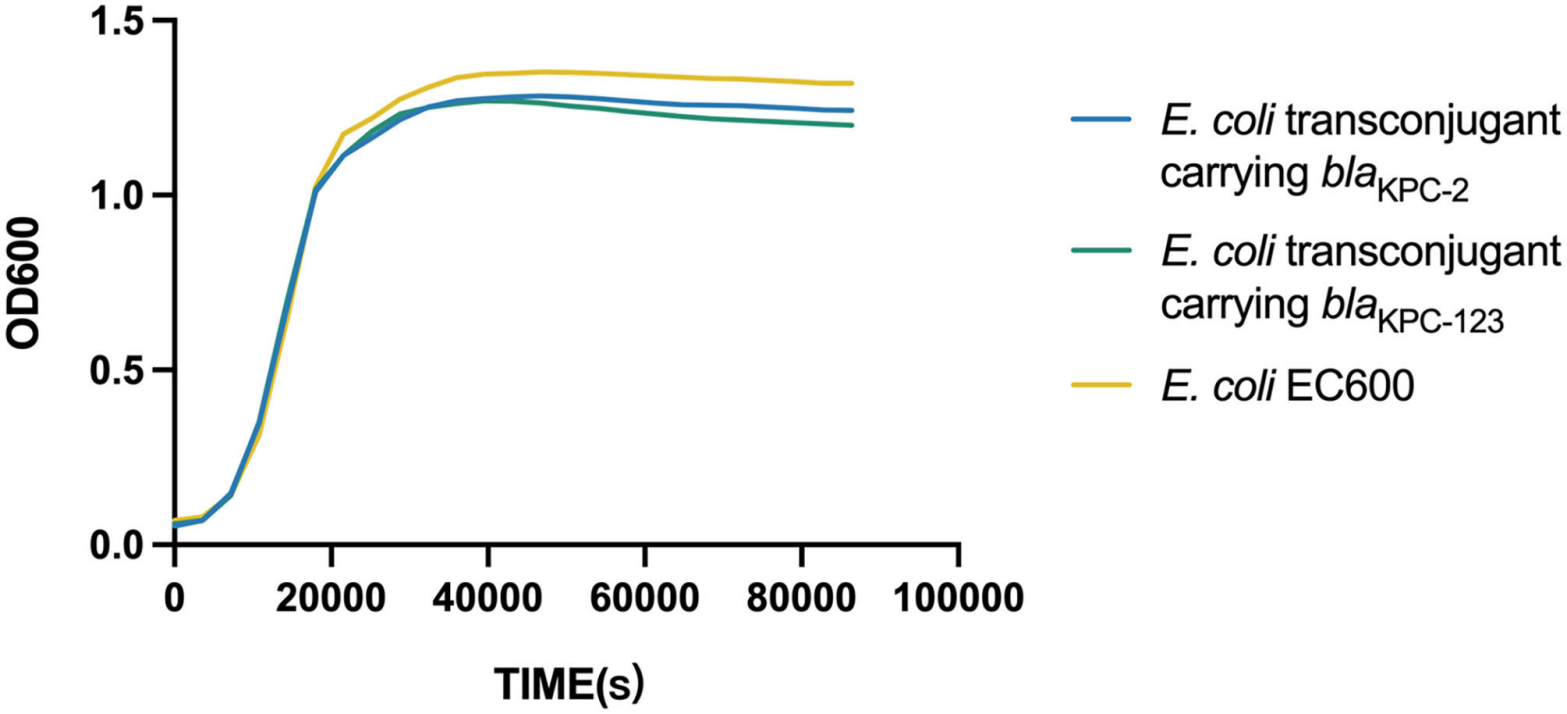
Growth curve of *E. coli* and the transconjugants carrying the *bla*_KPC_-bearing plasmid.

Growth curves were plotted and compared to evaluate the biological fitness for *E. coli* EC600 acquiring the *bla*_KPC−2_-carrying or *bla*_KPC−123_-carrying plasmid. Dislike the previously reported *bla*_KPC−71_ gene in China (Li et al., 2021a), no significant difference in the growth rates was observed among the *bla*_KPC−2_-positive transconjugant, the *bla*_KPC−123_-positive transconjugant, and the recipient strain (Figure 2). This result suggested that the acquisition of the *bla*_KPC−2_-carrying plasmid did not increase the fitness burden for the growth of *E. coli* EC600 in LB broth. Similarly, the mutations in KPC-2 did not affect the bacterial fitness.

## Discussion

**Figure 3:**
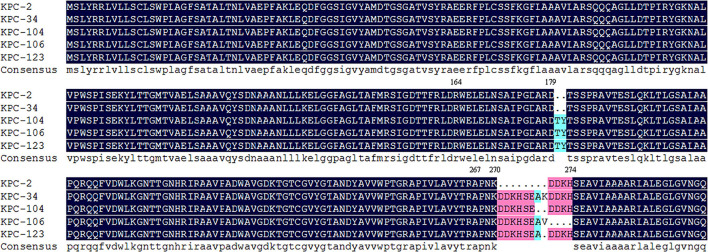
Sequence alignment of KPC-2 and KPC variants carrying similar mutations.

Ceftazidime-avibactam-resistant KPC variants rapidly increased in recent years (Wang et al., 2020). One hundred and thirteen KPC variants have been recorded in the Bacterial Antimicrobial Resistance Reference Gene Database of NCBI, 38 of which were inhibitor-resistant. Mutations conferring ceftazidime-avibactam-resistance mainly occurred in three “hot spots,” including the Ω-loop (position 164-179), Loop_238−243_, and Loop_267−275_, within KPC enzyme from both clinical isolates and mutants selected in the laboratory (Hobson et al., 2020; Wang et al., 2020). The novel KPC-123 reported here contained two inserts in two regions (ins179_TY and ins270_DDKHSEA). A similar mutation profile can be found by BLASTP search. KPC-104 (containing ins179_TY and ins270_DDKHSE, GenBank accession no. WP_231869651.1) and KPC-106 (containing ins179_TY and ins274_SEAV, GenBank accession no. WP_231869655.1) were identified in *K. pneumoniae* from Brazil while KPC-34 containing ins270_DDKHSEAK (GenBank accession no. WP_109545044.1) was found in *K. pneumoniae* from Taiwan, suggesting such natural mutants could occur independently under certain conditions (Figure 3). Our study showed that the long course of treatment with ceftazidime-avibactam might be involved.

The OXA-232-producing *K. pneumoniae*, which was domestically reported in Shanghai for the first time in 2016 (Yin et al., 2017), further contributed to another clonal dissemination among elderly patients in a hospital in Hangzhou 2 years later (Shu et al., 2019). Nowadays, such organisms have spread to several other cities in Zhejiang province. Moreover, clonally related isolates (*K. pneumoniae* WS420, CF503, and RS503) can be found in various samples of the same patient in this study, suggesting the high transmissibility of this ST15 OXA-232-producing *K. pneumoniae*. As a novel β-lactam/β-lactamase inhibitor combination with activity against CRE that produced OXA-48-like carbapenemase, ceftazidime-avibactam provides an effective therapeutic alternative against such organisms. However, the emergence and spread of mutants with ceftazidime-avibactam resistance under selection pressure cannot be ignored.

In China, the clinical use of antibiotics was strictly managed by the government laws and regulations. Some special antibiotics such as ceftazidime-avibactam, colistin, and tigecycline can only be prescribed by senior clinicians when necessary. There is an administration for rational use of antibiotics that is responsible for the review and approval of these antibiotics for clinical usage in our hospital. In some rare cases, such as severe and complex intracranial infection caused by CRKP in this study, last-resort antibiotics will be used for a long course of treatment, which may pose a risk to the *in vivo* evolution of antimicrobial resistance. Therefore, the establishment of an effective antimicrobial stewardship intervention and the constant surveillance of resistance development are of great significance.

## Conclusion

This study identified a novel KPC-123 β-lactamase that was resistant to ceftazidime-avibactam. We further depicted the possible evolution route of the *bla*_KPC_-123-carrying plasmid in *C. koseri* CK1008, in which the *bla*_KPC_-2-carrying plasmid originating from *K. pneumoniae* SP422 underwent mutational changes that conferred resistance to ceftazidime-avibactam under the prolonged exposure of this compound. Similarly, this ceftazidime-avibactam-resistant plasmid has the potential to horizontally transfer to other organisms, especially, CR-hvKP. Therefore, active surveillance of such plasmids is needed and may facilitate the prevention and control of the dissemination of ceftazidime-avibactam resistance.

## Data Availability Statement

The datasets presented in this study can be found in online repositories. The names of the repository/repositories and accession number(s) can be found in the article/Supplementary Material.

## Author Contributions

JC and RZ conceived and designed the work. JC collected and provided the isolates. LW and WS performed the experiments and analyzed the data. LW drafted the manuscript. All the authors revised the manuscript and approved the final version.

## Funding

The work was supported by the Zhejiang Provincial Natural Science Foundation of China (LY22H200001).

## Conflict of Interest

The authors declare that the research was conducted in the absence of any commercial or financial relationships that could be construed as a potential conflict of interest.

## Publisher's Note

All claims expressed in this article are solely those of the authors and do not necessarily represent those of their affiliated organizations, or those of the publisher, the editors and the reviewers. Any product that may be evaluated in this article, or claim that may be made by its manufacturer, is not guaranteed or endorsed by the publisher.
