# Supplementary material for: Identification of a Novel Ceftazidime-Avibactam-Resistant KPC-2 Variant, KPC-123, in Citrobacter koseri Following Ceftazidime-Avibactam Treatment
Source: Front Microbiol. 2022 Jun 20;13:930777. doi: 10.3389/fmicb.2022.930777 (PMC9251512; doi:10.3389/fmicb.2022.930777)
Supplement: Supplementary file 1 [file Data_Sheet_1.PDF]

Supplementary Figure S1. Phylogenetic analysis of four *K. pneumoniae* isolates in this study and 60 OXA-232-producing *K. pneumoniae* isolated from five hospitals in three cities of Zhejiang Province from 2018 to 2021. SAHZU, The Second Affiliated Hospital of Zhejiang University School of Medicine; SAHZU-BJ, The Second Affiliated Hospital of Zhejiang University School of Medicine-Binjiang Campus; HZXHH, The Second Affiliated Hospital of Zhejiang Chinese Medical University; HZTCMH, Hangzhou Traditional Chinese Medicine Hospital; JXH, Jiaxing people's Hospital; TZH, Tiazhou people's Hospital. ICU, intensive care unit; EICU, emergency intensive care unit; NICU, neurosurgical intensive care unit; SICU, surgical intensive care unit; BICU, burn intensive care unit. The KPC-2-producing *K. pneumoniae* SP422 was labelled as blue and three OXA-232-producing *K. pneumoniae* (strains WS420, CF503, and RS503) were labelled as red.

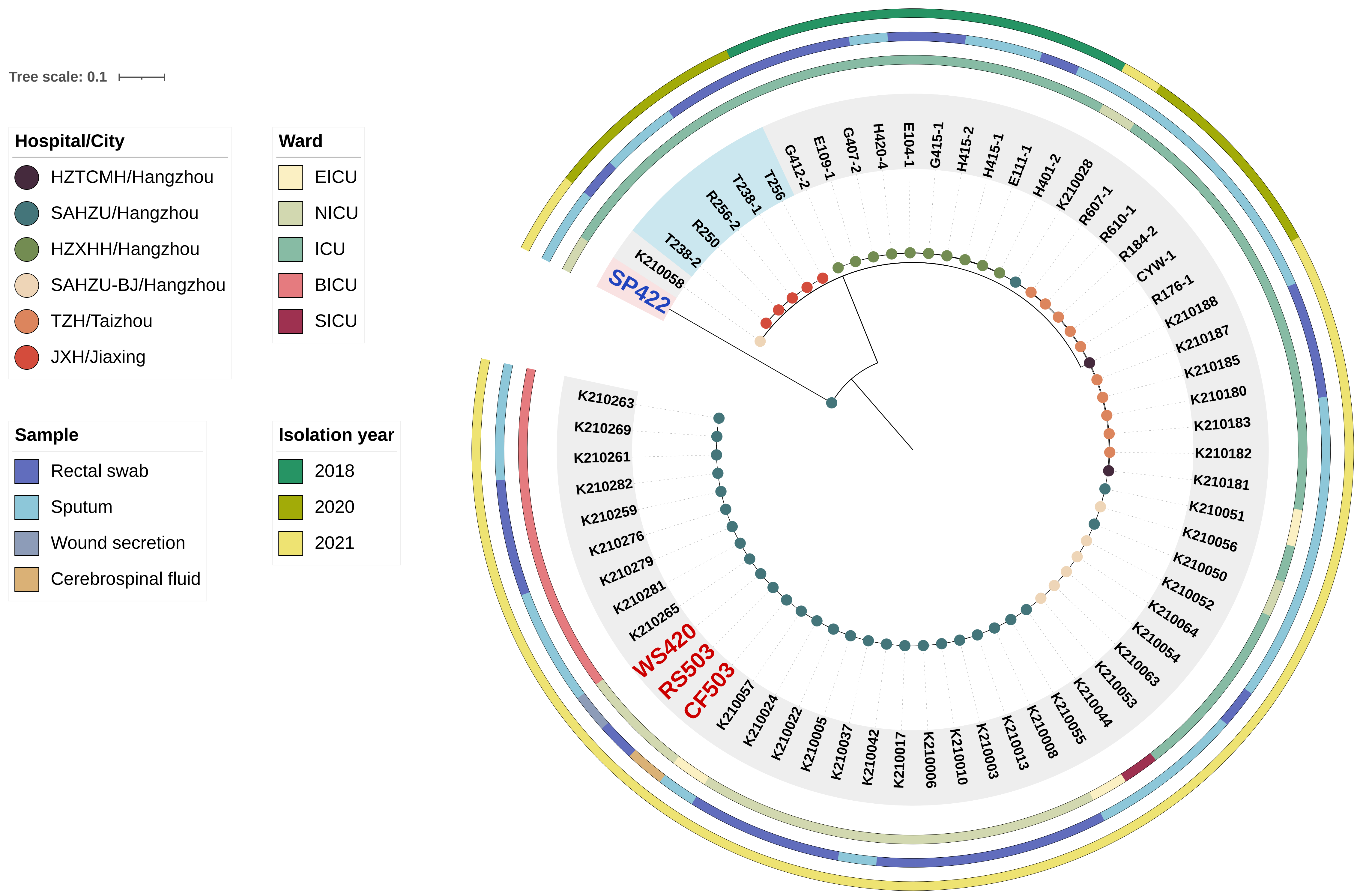
Tree scale: 0.1
Hospital/City
HZTCMH/Hangzhou
SAHZU/Hangzhou
HZXHH/Hangzhou
SAHZU-BJ/Hangzhou
TZH/Taizhou
JXH/Jiaxing
Ward
EICU
NICU
ICU
BICU
SICU
Sample
Rectal swab
Sputum
Wound secretion
Cerebrospinal fluid
Isolation year
2018
2020
2021
SP422
K210058
T238-2
R250
R256-2
T238-1
T256
G412-2
E109-1
G407-2
H420-4
E104-1
G415-1
H415-2
H415-1
E111-1
H401-2
K210028
R607-1
R610-1
R184-2
CYW-1
R176-1
K210188
K210187
K210185
K210180
K210183
K210182
K210181
K210051
K210056
K210050
K210052
K210064
K210054
K210063
K210053
K210044
K210055
K210008
K210013
K210003
K210010
K210006
K210017
K210042
K210037
K210005
K210022
K210024
K210057
CF503
RS503
WS420
K210265
K210281
K210279
K210276
K210259
K210282
K210261
K210269
K210263
